# Methylophiopogonanone A Protects against Cerebral Ischemia/Reperfusion Injury and Attenuates Blood-Brain Barrier Disruption *In Vitro*

**DOI:** 10.1371/journal.pone.0124558

**Published:** 2015-04-21

**Authors:** Mingbao Lin, Wei Sun, Wan Gong, Zhiyu Zhou, Yasi Ding, Qi Hou

**Affiliations:** 1 State Key Laboratory of Bioactive Substance and Function of Natural Medicines, Institute of Materia Medica, Chinese Academy of Medical Sciences & Peking Union Medical College, Beijing, China; 2 Clinical Laboratory Center, Beijing Friendship Hospital, Capital Medical University, Beijing, China; 3 College of Basic Medical Science, Zhejiang Chinese Medical University, Hangzhou, China; 4 College of Basic Medical Science, Jiangxi University of Traditional Chinese Medicine, NanChang, China; Texas A&M University Health Science Center College of Medicine, UNITED STATES

## Abstract

Methylophiopogonanone A (MO-A), an active homoisoflavonoid of the Chinese herb *Ophiopogon japonicus* which has been shown to have protective effects on cerebral ischemia/reperfusion (I/R) injury, has been demonstrated to have anti-inflammatory and anti-oxidative properties. However, little is known about its role in cerebral I/R injury. Therefore, in this study, by using a middle cerebral artery occlusion (MCAO) and reperfusion rat model, the effect of MO-A on cerebral I/R injury was examined. The results showed that MO-A treatment reduced infarct volume and brain edema, improved neurological deficit scores, reversed animal body weight decreases, and increased animal survival time in the stroke groups. Western blotting showed that MO-A suppressed MMP-9, but restored the expression of claudin-3 and claudin-5. Furthermore, transmission electron microscopy were monitored to determine the blood–brain barrier (BBB) alterations *in vitro*. The results showed that MO-A markedly attenuated BBB damage *in vitro*. Additionally, MO-A inhibited ROS production in ECs and MMP-9 release in differentiated THP-1 cells *in vitro*, and suppressed ICAM-1 and VCAM-1 expression in ECs and leukocyte/EC adhesion. In conclusion, our data indicate that MO-A has therapeutic potential against cerebral I/R injury through its ability to attenuate BBB disruption by regulating the expression of MMP-9 and tight junction proteins.

## Introduction

Ischemic stroke, a complex devastating disease, is caused by drastic disruption of cerebral blood flow, resulting in a deficiency of glucose and oxygen and triggering a multi-step pathophysiological ischemic cascade. Disruption of the blood—brain barrier (BBB) and successive brain edema formation are two of the basic pathological changes in cerebral ischemia/reperfusion (I/R) injury. Focal cerebral ischemia induces BBB integrity loss, which allows intravascular fluid and proteins to penetrate into the cerebral extracellular space, thereby incurring vasogenic edema formation and further brain damage [1]. The BBB is a highly selective permeability barrier to separate the circulating blood from the brain extracellular fluid and to maintain the micro-environment of brain, which being formed of endothelial cells, pericytes, astrocytes, neurons, and the extracellular matrix [2]. Considerable evidence indicates that I/R oxidative stress can cause BBB disruption and increase cerebral vascular permeability, which then leads to the formation of brain edema, which aggravates cerebral infarction [3]. A sudden supply of molecular oxygen after reperfusion subsequently produces excessive reactive oxygen species (ROS) via electron transport within mitochondria in brain tissue. ROS then activates enzymes and signal cascades such as lipids and chromatin, activating matrix metalloproteinases (MMPs), changing tight junction proteins, and eventually resulting in BBB dysfunction [4, 5]. Therefore, protection of the BBB can thus be seen as an aspect of stroke management, by rescuing the neurovascular unit.

**Fig 1:**
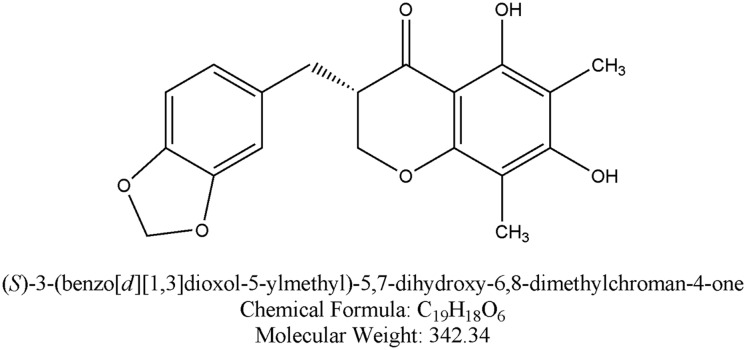
Chemical structure of Methylophiopogonanone A (MO-A).

Quite a few neuroprotective agents with moderate efficacy in stroke management have been reported. However, the treatment of stroke remains considerably unsatisfactory as the performance of almost all neuroprotective agents in the clinical treatment of patients with stroke has been disappointing [6–8]. Recent attention has focused on a series of natural products shown to protect against cerebral I/R injury. The dried tuber root of *Ophiopogon japonicus* (L.f) Ker-Gawl from *Ophiopogon japonicus plants*, which commonly being classified into Chuanmaidong and Zhemaidong according to its mainly producing areas of Sichuan and Zhejiang provinces in China, is one of the commonly traditional Chinese medicine in clinic, being used for the treatment of myocardial ischemia and thrombosis, remedying hypoxia, delaying senility and reducing blood sugar etc. for thousands of years [9]. It has been reported that a Chinese patent drug consisting of *Ophiopogon japonicus* (Shengmai San) might significantly protect the brain against I/R injury [10]. The main chemical constitutes of *Ophiopogon japonicus* involve steroidal sapogenins and homoisoflavonoids [9]. A major steroidal sapogenin of *Ophiopogon japonicus* (Ruscogenin) has been shown to prevent cerebral I/R injury [11], however, little is known about homoisoflavonoids of *Ophiopogon japonicus*. Methylophiopogonanone A (MO-A; Fig 1) is one of the major isoflavonoid present in *Ophiopogon japonicus*. Previous pharmacological investigations have revealed that both MO-A has anti-oxdative [12, 13] and anti-inflammatory properties [12]. However, to our knowledge, there are no reports about the effect of MO-A on cerebral I/R injury.

Therefore, in this study, rats subjected to middle cerebral artery occlusion (MCAO) and an *in vitro* model of BBB dysfunction were used to examine the therapeutic potential of MO-A on stroke prevention. Additionally, we assessed whether these cerebral protective effects were associated with inhibition of BBB disruption via regulating MMP-9 and claudin protein expression.

## Materials and Methods

Our findings were reported in accordance with the Animals in Research: The ARRIVE (Animal Research: Reporting In Vivo Experiments) Guidelines [14, 15].

### Transient Focal Cerebral Ischemia in the Rat

The experimental protocol was approved by the Ethics Committee for Animal Experimentation of Capital Medical University (Beijing, China) and conformed to internationally accepted ethical standards. Sprague-Dawley rats (Male, 280–320g weights) were supplied by the Vital River Laboratories (Beijing, China) and housed under controlled conditions with a 12-hour light/dark cycle, at a temperature of 21°C ± 2°C, and humidity of 60 ± 5% for at least 1 week before experimentation. The rats were allowed free access to a standard rodent diet and tap water.

MCAO was induced using the intraluminal suture method [16, 17]. Briefly, rats were anesthetized using chloral hydrate (400 mg/kg), which shown advantages of fast absorption, short excited periods and long periods of anesthesia (1–3 hours), while its possible side effects, such as strong irritant to local tissues, inhibitation of themoregulatory center and arrhythmia, should be caution; a 2.0-cm skin incision was made in the ventral neck muscles, and a 3–0 MCAO monofilament (Sunbio Biotech Co., Ltd., Beijing, China) was advanced to block the origin of the MCA. Regional cerebral blood flow (rCBF) was monitored using a laser Doppler computerized main unit (Perimed AB, Sweden). Successful establishment of MCAO was determined when rCBF decreased to 20% of baseline levels before ischemia; otherwise, animals were excluded. Reperfusion was performed by withdrawing the monofilament after 120 minutes ischemia, and then the wound was closed. During the surgery and reperfusion, rectal temperature was maintained at 37 ± 0.5°C by means of a heating blanket. Rats were examined once every 3 hours at day 1 and once every 12 hours at day 2 to day 7. “Low mobility” and “animals incapable of feeding” were used as humane endpoints. The mortality rate of the MCAO model was around 15%, while about 9% in the young rats and 43.5% in the aged rats [18]. A similar procedure without MCAO was conducted in control rats.

### Drug Treatments

To evaluate the neuroprotective effect of MO-A (purity > 98%; Ronghe Technology Co., LTD, Shanghai, China, and was dissolved in normal saline with PH = 8.0), except for sham operation rats (Control), MCAO rats were randomly divided into 4 groups: MCAO with vehicle (model), and MCAO treated with 1.25, 2.50 or 5.00 mg/kg MO-A twice per day. After 2 h reperfusion, the rats were treated intravenously with MO-A for 7 days. Control rats were treated with equal volumes of saline.

### Neurological Deficit Evaluation

Neurological function was evaluated blindly as described previously [19] at 6 hours, 1, 3 and 7 days after reperfusion. On a five-point scale (grade 0: showing no observable deficit; Grade 1: a failure to fully extend left forepaw; Grade 2: circling to the left; Grade 3: falling to the left; Grade 4: no walking spontaneously and having a depressed level of consciousness). The evaluation was performed by an observer who was blind to the group.

### Infarct Volume Measurement

On day 7 after MCAO, animals were euthanized and sacrifices by decapitation, and the brains were collected. Brains were sliced into 7 coronal sections at 2-mm thickness each and stained with 2% (w/v) 2,3,5-triphenyltetrazolium chloride (TTC; Sigma-Aldrich, USA) in saline at 37°C for 30 minutes, and then fixed in 4% (w/v) paraformaldehyde [16, 17]. Afere 24 hours, slices were scanned and images were analyzed with Image J 1.42q software (National Institutes of Health, USA), the normal tissue was stained with a deep red while a pale gray color in the infarct area, infarct volumes was expressed as percentage of the two fold of the contralateral hemisphere.

### Brain Water Content Determination

Animals were anesthetized and sacrificed by decapitation after reperfusion at 3 days. The brains were quickly removed and weighed to obtain the wet weight, and dried at 100°C for 24 h and weighed to obtain the brain water content. The formula for calculating water content was as follows: water content (%) = (wet weight − dry weight) / wet weight ×100.

### Western Blotting

Protein was extracted from the cortex according to a previously described procedure [20]. Membranes were probed with primary antibodies against claudin-3, claudin-5 (1:1000, Millipore, USA) and MMP-9 (1:1000, Cell signaling, USA). Horseradish peroxidase-conjugated anti-rabbit or anti-mouse secondary antibodies were used and visualized using the enhanced chemiluminescence kit (Thermo Scientific, USA). The density of each band was quantified with Image J 1.42q software (National Institutes of Health, USA). β-actin (1:5000, Abmart, China) was used as a loading control.

### Cell Culture and Treatment

Mouse brain endothelial bEND.3 cells (American Type Culture Collection, Manassas, VA, USA) were cultured in Dulbecco’s modified Eagle’s medium (4500 mg/L glucose) supplemented with 10% (v/v) fetal bovine serum at 37°C in a humidified atmosphere of 5% (v/v) CO_2_. Cells were seeded at 5×10^5^ cells per well on 24 well plates. After 12 hours, cells were incubated in a hypoxia chamber (Changjin Institute of Applied Technology, China) with Dulbecco’s modified Eagle’s medium (no glucose) supplemented with 10% (v/v) fetal bovine serum at 37°C in a 5% (v/v) CO_2_/ 95% (v/v) N_2_ for 12 hours, and then incubated under normal conditions at 37°C for another 24 hours. Before hypoxia, cells were treated with MO-A (2.5, 5.0 or 10 μM), and control cells were cultured under normal conditions with vehicle. After stimulation, cells were lysated with an ultrasonic lysis instrument (Sonics, USA), and supernatants were collected for ICAM-1 and VCAM-1 ELISA detection.

Human monocytic THP-1 cells (American Type Culture Collection) were cultured in RPMI 1640 medium supplemented with 10% (v/v) fetal bovine serum at 37°C in a humidified atmosphere of 5% (v/v) CO_2_. Cells were seeded at 5×10^5^ cells per well on 24 well plates, and stimulated with PMA (200 nM) or PMA (200 nM) + MO-A (2.5, 5.0 or 10 μM) for 24 hours. After stimulation, cell culture supernatants were collected for MMP-9 ELISA detection, the MMP-9 ELISA was performed according to the protocols of human MMP-9 quantikine ELISA kit (DMP900, R&D Inc.).

### Cell viabilities were determined with MTT assay

Cell viabilities were measured using the MTT assay. bEnd.3 cell were seeded in 96-well plates at concentrations of 1×10^4^ cells per well. After incubation for 12h, the cells were OGD incubated for 12 hours with MO-A treatment, and then incubated under normal conditions with MO-A treatment at 37°C for another 24 hours. Following the washing step, 20 μL of MTT solution (5 mg/ml in PBS) was added to each well, and the cells were incubated at 37°C for another 4 hours. Finally, the culture medium was removed and 150 μL of DMSO was added. Absorbances at 570 nm were measured using amicroplate reader (BioTek, Winooski, VT, USA).

### Measurement of Transendothelial Electrical Resistance

bEND.3 cells were grown to confluence on fibronectin coated cell inserts (Millipore, USA) with 8 μm pore size, and incubated in a hypoxia chamber (Changjin Institute of Applied Technology, China) with a 5% (v/v) CO_2_/ 95% (v/v) N_2_ at 37°C for 12 hours, and then incubated under normal conditions at 37°C for another 24 hours. Before hypoxia, cells were treated with MO-A (2.5, 5.0 or 10 μM) for 24 hours. Control cells were cultured under normal conditions with vehicle. The resistance of inserts was measured using the Millicell ERS Voltohmmeter (Millipore).

### Measurement of Reactive Oxygen Species

After treatments with MO-A for 12 hours, intracellular ROS generation was measured using 2, 7-dichlorofluorescein diacetate (DCF-DA) fluorescent dye. Following hypoxia/re-oxygenic incubation, bEnd.3 cells were loaded with DCF-DA (5 μmol/L) for 30 minutes at 37°C, and then, were imaged by confocal microscopy (Leica, Germany). Fluorescent images of 3 different fields (0.81 mm^2^) randomly selected per well were obtained, and mean fluorescent intensity of the images was measured.

### Leukocyte/EC Adhesion

The adhesion of Leukocyte to EC was examined under static conditions as described previously [21]. Briefly, THP-1 cells were labeled with calcein red-orange-acetoxymethyl ester at 1 μmol/L for 10 minutes at 37°C. Following stimulation, bEnd.3 were labeled with calcein/AM at 1 μmol/L for 10 minutes at 37°C, followed by co-incubation with THP-1 cells (5×10^5^ cells per well) for 30 minutes at 37°C. Nonadherent THP-1 cells were then removed by washing with phosphate buffered saline (PBS). Subsequently, THP-1 cells adhering to the bEnd.3 monolayer were imaged by confocal microscopy (Leica, Germany). The mean density of adherent cells was determined by counting the number of THP-1 cells in 3 different fields (1.8 mm^2^).

### Statistical Analysis

Statistical analyses were performed with SPSS 11.5 Statistical software (SPSS Inc.) and statistical significance was set at P < 0.05 or P < 0.01. Data was presented as the mean ± SD. As the normality test by Kolmogorov-Smirnov test (K-S test) was passed, data was analyzed by using the Student’s *t*-test for comparison between two groups and one-way ANOVA for multiple groups followed by Fisher’s least significant difference (LSD) test, otherwise, by using Kruskal-Wallis H test.

## Results

### MO-A Protects against Cerebral I/R Injury and brain edma

**Fig 2:**
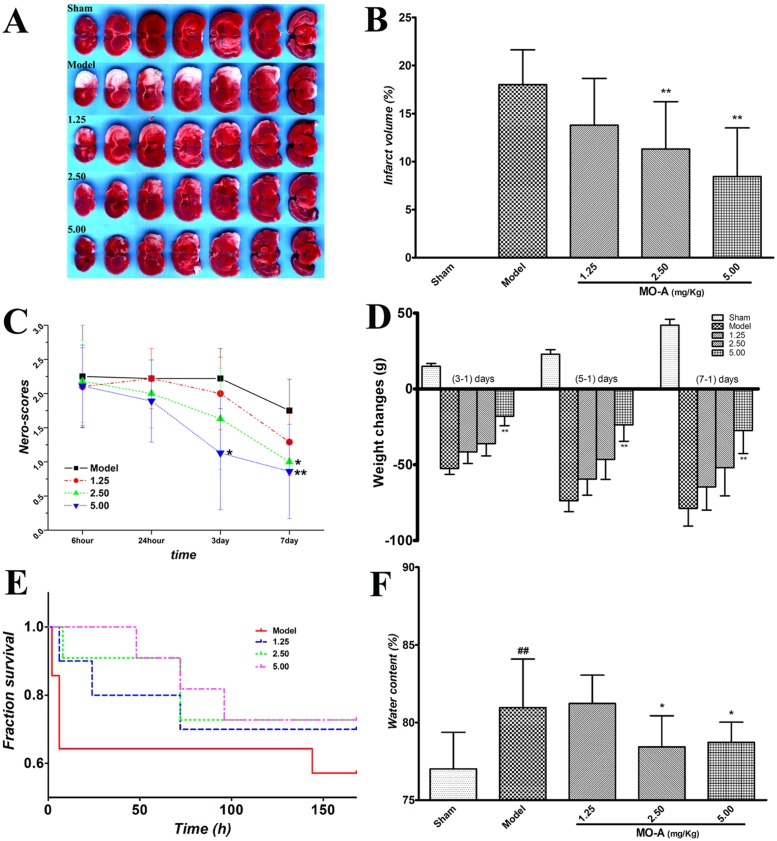
MO-A protects against cerebral injury after MCAO in rats. (A) Infarct images using TTC staining at day 7 after MCAO; (B) Determination of infarct volume at day7 after MCAO (n: sham = 14, model = 9, MO-A 1.25mg/Kg group = 8, 2.50 mg/Kg group = 7, 5.00 mg/Kg group = 8); (C) Neurological deficit was evaluated using a five-point scale (n = 6–14); (D) The changes of animal body weights at day 3, 5 and 7, subtracted the body weights of day 1 (n = 6–14); (E) Animal survival times, rats were examined once every 3 hours at day 1 and once every 12 hours at day 2 to day 7 (n = 6–14). “Low mobility” and “animals incapable of feeding” were used as humane endpoints; (F) Determination of brain water contents in transient MCAO rats (n = 6–14); Data are given as mean ± S.D.; ^#^ *P* < 0.05 and ^##^ *P* < 0.01 *vs*. sham group by Student’s *t*-test statistical analysis; *P < 0.05 and **P < 0.01 *vs*. MCAO model group by Kruskal-Wallis H test in 7 day neurological deficit (C), weitht changes of 7 day (D) and water contents (F), and others by one-way ANOVA statistical analysis.

The infarct area of each treatment group was observed by TTC staining (Fig 2A). Infarct volume was analyzed, and the results showed that there was no infarct in sham animals, but significant ischemic injury following transient MCAO (model group). TTC staining revealed that MO-A (2.5 mg/kg, 5.0 mg/kg) significantly reduced infarct volume when compared with the model group (Fig 2B). Following treatment with MO-A, the infarct volume was reduced in a dose-dependent manner.

After reperfusion, neurological scores were measured at 6 hours, 24 hours, 3 days and 7 days (Fig 2C), and animal body weights were measured at day 1, 3 and 7 (Fig 2D). The results showed that neurological scores of MCAO animals with MO-A treatment at a dose of 2.5 mg/kg and 5.0 mg/kg were significantly decreased when compared with the model group (P < 0.05 or 0.01). Additionally, the decrease in animal body weights was significantly reversed with MO-A (5.0 mg/kg) treatment (P < 0.01).

Furthermore, animal survival time was also observed to evaluate the protective effects of MO-A in MCAO rats. Although no significant differences were observed, the results showed that the survival time of MCAO animals with MO-A treatment (127.8 ± 66.7, 136.0 ± 57.2 and 141.8 ± 46.1 hours of 1.25, 2.50 or 5.00 mg/kg dosage treatment, respectly) increased when compared with the model group (107.9 ± 80.3 hours) (Fig 2E).

Additionally, To investigate the protective effects of MO-A on BBB disruption under hypoxic conditions, the brain water contents in MCAO rats were measured. As shown in Fig 2F, cerebral water contents were less in the MO-A (2.5 mg/kg and 5.0 mg/kg) treatment groups than those in the model group (P < 0.05).

### MO-A protects against oxygen-glucose deprivation/reperfusion (OGD/R) induced brain endothelial cell injury and prevents BBB disruption *in vitro*

**Fig 3:**
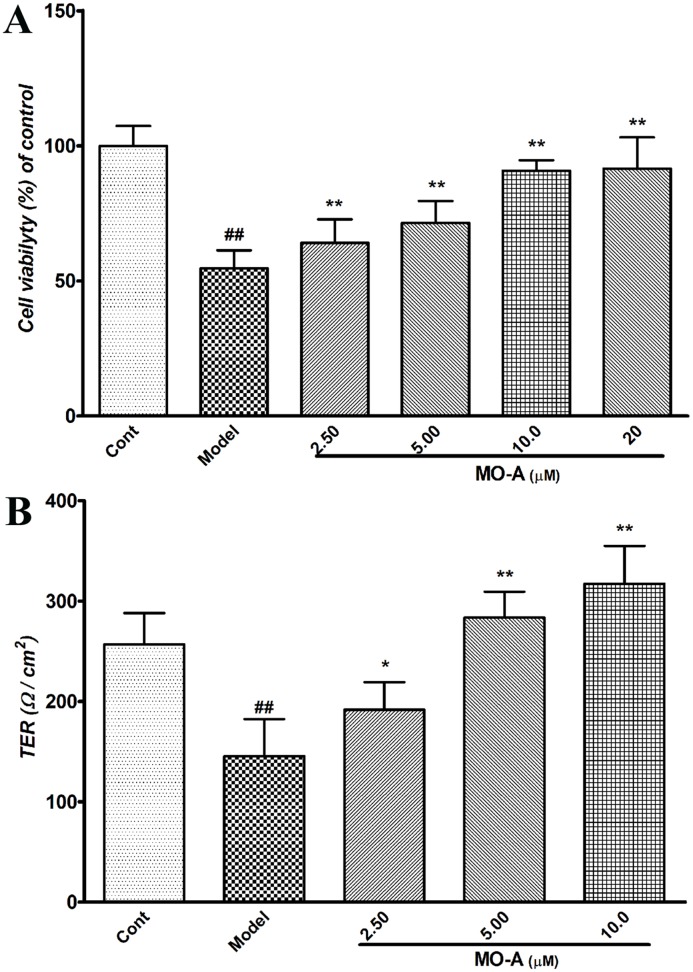
Effect of MO-A on BBB disruption. (A) cell viability of OGD/R-induced bEnd.3 cell injury was determined with MTT assay; (B) Effect of MO-A on hypoxic BBB damage *in vitro*; Data are given as mean ± S.D. (n = 3); ^#^ *P* < 0.05 and ^##^ *P* < 0.01 *vs*. sham group by Student’s *t*-test statistical analysis; *P < 0.05 and **P < 0.01 *vs*. MCAO model group by one-way ANOVA statistical analysis.

To investigate the protective effects of MO-A on BBB disruption under hypoxic conditions, the transendothelial electric resistance (TER) of a hypoxic BBB damage model *in vitro* was measured. As showed in Fig 3A, MO-A treatment in vitro significantly improved the cell survival in OGD/R induced cell injury. Furthermore, *in vitro*, barrier tightness in bEnd.3 monolayers was markedly compromised under hypoxic/reoxygenic conditions (model), as reflected by a significant decrease in TER when compared with monolayers maintained under normoxic conditions (control). Interestingly, when compared with the model group, the decreased TER values were significantly reversed by MO-A treatment, which indicated that MO-A had a protective effect on hypoxic BBB damage (Fig 3B).

### MO-A suppresses the expression of MMP-9, and reverses the reduction of claudin-3 and claudin-5 expression in rats after transient MCAO

**Fig 4:**
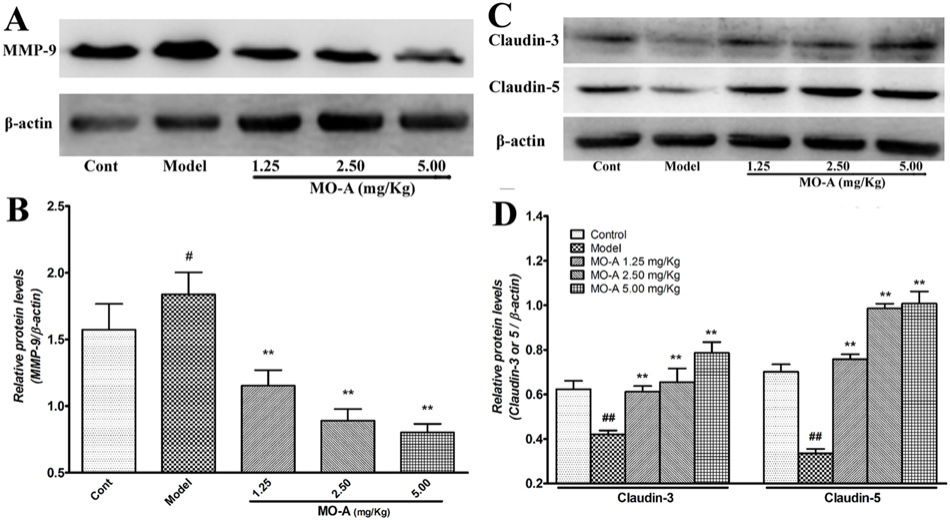
Effect of MO-A on the expression of MMP-9 and tight junction proteins after MCAO in rats. (A) Representative blots of MMP-9 determined by Western blotting; (B) Quantitative analysis of the ratio of MMP-9; (C) Representative blots of claudin-3 and claudin-5 determined by Western blotting; (D) Quantitative analysis of the ratio of claudin-3 and claudin-5; Data are given as mean ± S.D. (n = 3); ^#^ *P* < 0.05 and ^##^ *P* < 0.01 *vs*. sham group by Student’s *t*-test statistical analysis; *P < 0.05 and **P < 0.01 *vs*. MCAO model group by one-way ANOVA statistical analysis.

Western blotting analysis revealed that I/R significantly upregulated the expression of MMP-9 in the model group when compared with the sham group. Meanwhile, the administration of MO-A significantly decreased the expression of MMP-9 when compared with the MCAO model group (Fig 4A and 4B).

Furthermore, as shown in Fig 4C and 4D, I/R markedly diminished the expression of claudin-3 and claudin-5 when compared with the sham group. Interestingly, the reduced expression levels of claudin-3 and claudin-5 caused by I/R injury were significantly reversed by MO-A treatment.

### MO-A reduces OGD/R-induced ROS generation in bEnd.3 cells

**Fig 5:**
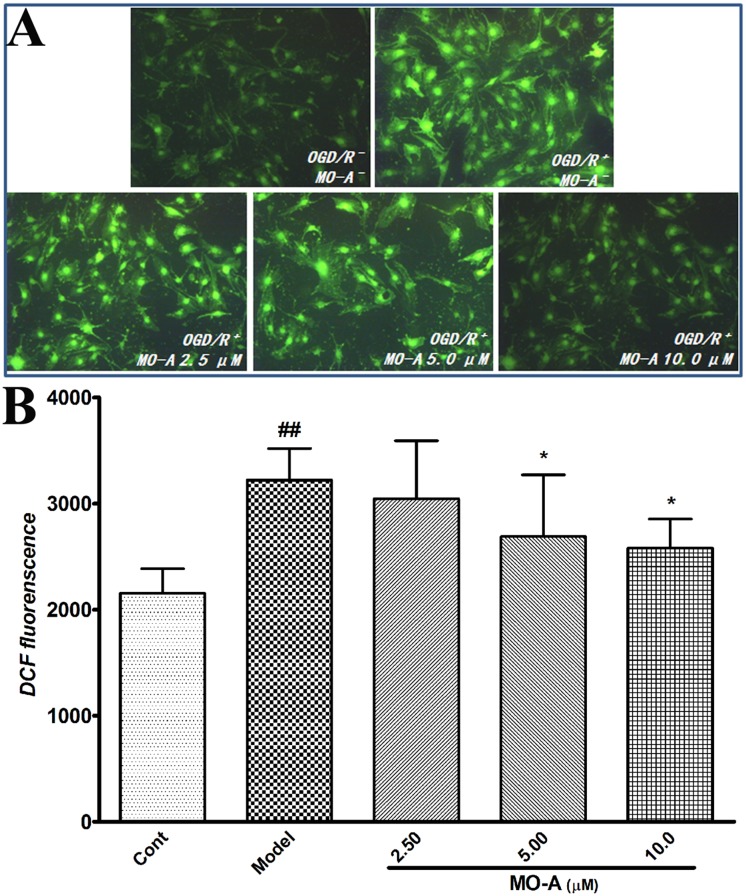
Effects of MO-A on OGD/R-induced ROS generation in bEnd.3 cells. (A) DCF images indicate ROS generation in bEnd.3 cells; (B) graph represents quantification of fluorescence intensity in the images; Data are given as Mean ± S.D. (n = 6), ^#^ *P* < 0.05 and ^##^ *P* < 0.01 *vs*. sham group by Student’s *t*-test statistical analysis; *P < 0.05 and **P < 0.01 *vs*. MCAO model group by one-way ANOVA statistical analysis.

Previous observations indicated that BBB disruption involves the generation of ROS and oxidative injury in brain vascular endothelial cells [21, 22]. To study the anti-oxidative effects of MO-A, intracellular ROS levels were analyzed in bEnd.3 cells using the ROS-detecting fluorescent dye DCF (Fig 5). DCF analysis showed that OGD/R significantly induced an increase in ROS formation in bEnd.3 cells when compared with the normoxic control, while treatment with MO-A significantly abolished this increase in ROS when compared with the OGD/R groups.

### Direct Effects of MO-A on the activation of endothelial cells and leukocytes

**Fig 6:**
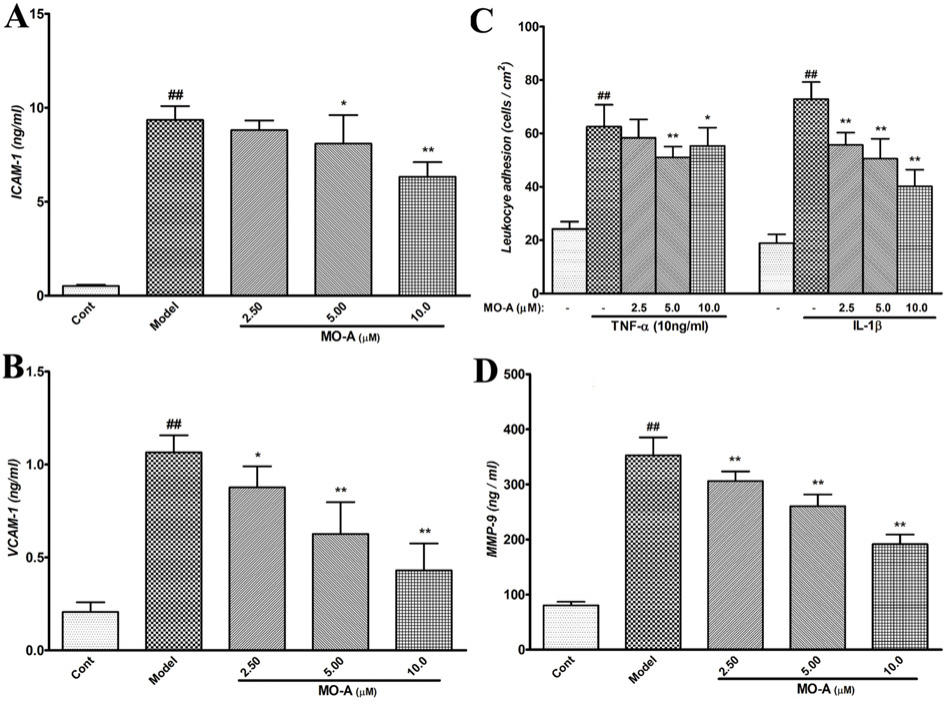
Direct effects of MO-A on the activation of endothelial cells and leukocytes. (A) Expression of ICAM-1 in OGD/R-induced bEnd.3 cells using ELISA, data are shown as Mean ± S.D. (n = 6); (B) Expression of VCAM-1 in OGD/R-induced bEnd.3 cells using ELISA, data are shown as Mean ± S.D. (n = 6); (C) Density of THP-1 cell adhesion to bEnd.3 following stimulation by TNF-α (10 ng/mL) or IL-1β (10 ng/ml), data are shown as Mean ± S.D. (n = 3); (D) MMP-9 secretion in undifferentiated and differentiated THP-1 cells stimulated by PMA (200 ng/mL) as detected by ELISA, data are shown as Mean ± S.D. (n = 6); ^#^ *P* < 0.05 and ^##^ *P* < 0.01 *vs*. sham group by Student’s *t*-test statistical analysis; *P < 0.05 and **P < 0.01 *vs*. MCAO model group by one-way ANOVA statistical analysis.

As shown in Fig 6A and 6B, the level of ICAM-1 and VCAM-1 were significantly enhanced in OGD/R-induced bEnd.3 cells (model group) when compared with the control group. MO-A treatment significantly inhibited the increase of ICAM-1 and VCAM-1 induced by OGD/R-treatment.

Next, we performed an adhesion assay using THP-1 cells and bEnd.3 cells in the presence of TNF-α and IL-1β (Fig 6C). Adhesion of leukocytes to the endothelial cell layer was significantly increased when compared with cells not treated with TNF-α or IL-1β, and the increase was markedly inhibited with MO-A treatment.

Furthermore, MMP-9 release in differentiated THP-1 cells were detected by ELISA (Fig 6D). The results showed that the secretion of MMP-9 in THP-1 cells was significantly enhanced after 24-hour phorbol-12-myristate-13-acetate (PMA, Sigma-Aldrich, USA) treatment when compared with untreated cells, and this increase was markedly prevented with MO-A treatment (P < 0.01).

## Discussions

MCAO rats, when administered with MO-A at 2.50 and 5.00 mg/kg intravenously twice per day, had significantly reduced infarct volumes following TTC staining, and significantly alleviated the neurological deficits and body weight loss of the rats with MCAO, which indicating that the less histological damage was observed after MO-A treatment. It suggests that MO-A has significant therapeutic potential on brain ischemic injury.

Mechanical or hypoxic damage of vascular endothelium, toxic damage of excitotoxic amino acids (especially glutamate), inflammatory molecules and free radicals, and destruction of the basal lamina by matrix metalloproteinases are potential causes of BBB disruption, which leads to vasogenic edema, influx of toxic substances and inflammation after stroke [23]. Vasogenic edema, being distinct from cytotoxic edema, occurs due to a breakdown of the tight endothelial junctions that make up the blood–brain barrier, which allows intravascular proteins and fluid to penetrate into the parenchymal extracellular space [24]. In the present study, we demonstrated that MO-A reduced water content after reperfusion when compared with the model group, which suggested that MO-A had a protective effect on ischemic brain edema. We also demonstrated that I/R impaired BBB permeability BBB while MO-A treatment restored BBB integrity following hypoxic BBB damage *in vitro*. This result indicates that the protective effect of MO-A on brain ischemic injury involves its ability to attenuate BBB disruption *in vitro* and brain vasogenic edema.

Brain capillary endothelial cells expressing tight junction proteins are the major structural constituents of the BBB [25]. Degradation of the critical tight junction proteins claudin-3 and claudin-5 is involved in BBB breakdown and a pathological hallmark of acute ischemic stroke [26, 27]. This degradation of tight junction proteins was involved in triggering numerous molecular cascades that suppress MCAO-induced over expression of MMP-9 [28, 29]. MMP-9 has been extensively studied for its involvement in BBB disruption after stroke [30]. It has been reported that MMP-9 knockout mice are resistant to BBB disruption induced by transient focal cerebral ischemia [31]. Accordingly, in this study, we observed robust upregulation of MMP-9 in the brain after transient MCAO, which was significantly decreased with MO-A treatment. We also found that I/R altered the expression of tight junction proteins (claudin-3 and claudin-5), while MO-A treatment normalized their expression. These results suggest that the protective effect of MO-A on BBB disruption may associated with the inhibition of MMP-9 and regulation of tight junction proteins.

MMP-9 over expression has been shown to be strongly associated with oxygen radicals [32]. Reactive oxygen species, as a consequence of focal cerebral ischemia and reperfusion, are produced under ischemic conditions and during the inflammatory response after ischemia [33], which react irreversibly with several cellular constituents and damage virtually any cellular component [23]. Oxygen radicals serve as important signaling molecules in the pathophysiology of BBB damage and ischemic stroke [23]. Oxygen radicals not only triggers the activation of MMP-9 in brain I/R injury [29], but also elicits an inflammatory response and plays a key role in the induction of cell adhesion molecules [21]. Several studies have shown that cerebral infarction induced by transient MCAO is significantly reduced when leukocyte adhesion to ECs is inhibited [34, 35]. In this study, we found that treatment with MO-A may significantly abolish the increase of ROS and the level of ICAM-1 and VCAM-1 in OGD/R bEnd.3 cells, followed by marked inhibition of the adhesion of leukocytes to endothelial cells in the presence of TNF-α and IL-1β. Additionally, MMP-9 release in PMA differentiated THP-1 cells was also significantly prevented with MO-A treatment. These results suggest that the inhibition of MMP-9 release and leukocyte adhesion to ECs may be associated with the inhibition of ROS increase.

Collectively, these data clearly indicate that the protective effect of MO-A on cerebral I/R injury partly involves its ability to attenuate BBB disruption *in vitro* and brain edema. The potential mechanism of this BBB disruption attenuation following MO-A treatment may be associated with the regulation of tight junction proteins and MMP-9, which is likely to be mediated by attenuating oxidative stress, and the resultant inhibition of leukocyte and EC inhibition.

## Supporting Information

S1 ARRIVE Checklist The animal research reporting in vivo experiments were checked by using the ARRIVE Guidelines Checklist. (PDF)
